# Characteristic of complete mitochondrial genome and phylogenetic status of a muskrat, *Ondatra zibethicus* (Rodentia: Cricetidae) in South Korea

**DOI:** 10.1080/23802359.2020.1768916

**Published:** 2020-05-22

**Authors:** Young-Chae Kim, Do-Hun Lee, Hae-Jun Baek, Areum Kim, Jemin Lee, Min-Han Kim, Yong-Ho Kwon, Yung Kun Kim

**Affiliations:** a Restoration Research Team (Mammals), Research Center for Endangered Species, National Institute of Ecology, Gyeongsangbuk-do, Korea; b Invasive Alien Species Research Team, Bureau of Survey and Safety Research, National Institute of Ecology, Chungcheongnam-do, Korea; c Gyeryongsan National Park Office, Chungcheongnam-do, Korea

**Keywords:** *Ondatra zubethicus*, mitochondrial genome, Rodentia

## Abstract

The complete mitochondrial genome of Muskrat, *Ondatra zibethicus* (Rodentia: Cricetidae) in Korea was sequenced for the first time using the next-generation sequencing method to understand its evolutionary relationship and to be helpful to establish a management plan. This mitogenome was 16,350 base pairs in length, containing 13 protein-coding genes, 22 transfer RNA genes, two ribosomal RNA genes, and one control region. Its overall A, C, G, and T contents were 32.0%, 26.9%, 12.6%, and 28.5%, respectively. A + T content (63.7%) was higher than G + C content (36.3%). We made the phylogenetic tree of muskrat and other 12 species of order Rodentia distributed in Korea.

The muskrat, that belongs to Order Rodentia, Family Cricetidae, is native to North America including northern Canada, the United States, and Mexico (Willner et al. 1980; Ceballos 2014). It was introduced to various regions around the world for the economic benefits, such as fur industry, since the early 20th century, but a decline in the economic benefits of the breeding industry due to deterioration of fur usage has led to the lack of management of the introduced animals and has since been known to have settled in natural ecosystems in several countries (Niethammer and Krapp 1982; Long 2003; Neronov et al. 2008; IUCN 2016). As an alien species, muskrats have various damage and negative effects on the ecosystem of the countries where they have introduced artificially (Skyrienė and Paulauskas 2013).

This study was conducted with the purpose of establishing a management plan for Muskrat that has settled in the ecosystem of Korea and caused various damages, originally. In addition, genetic analysis was executed because enlightening the history of genetic evolution of Muskrat in Korea was essential to establishing a management plan.

In this study, we could get a carcass of *O. zibethicus* near a muskrat farm (N36°35’11.81”, E127°18’29.77”), while we were investigating. The specimen had been kept in the National Institute of Ecology at −80 °C until the genomic DNA was extracted (Specimen ID OZ-003). We extracted genomic DNA using Qiagen DNeasy Blood and Tissue kit (Qiagen Korea Ltd, Seoul, South Korea) following the manufacturer’s instruction with minor modification. And, the complete mitochondrial DNA sequence was analyzed on a Hiseq2000 platform using Next-Generation Sequencing (NGS) technique (Illumina, San Diego, CA). tRNA Scan-SE 1.21 (http://lowelab.ucsc.edu/tRNA Scan-SE/) was used to search for tRNA genes in genomic sequences. Geneious 10.2.6 (Biomatters Ltd, Auckland, New Zealand) and MITOS WebServer (http://mitos.bioinf.uni-leipzig.de) were used to assemble and annotate the mitochondrial DNA sequences. Phylogenetic tree that shows the comprehensive evolutionary relationship among other species of order Rodentia was constructed using MEGA-X based on maximum likelihood analysis with GTR model (General Time Reversible model) (Figure 1) (Kumar et al. 2018).

**Figure 1.**
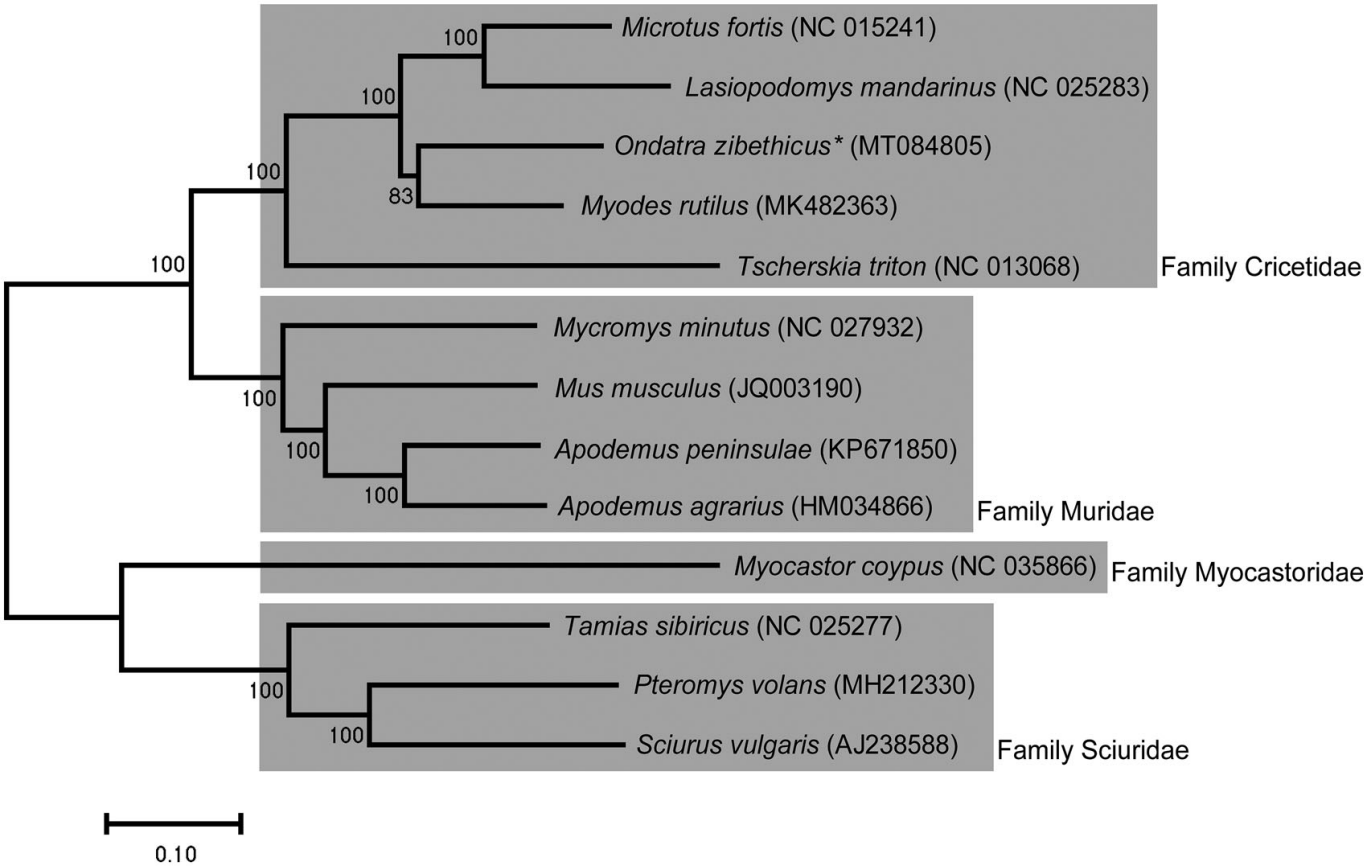
Maximum-likelihood phylogenetic tree of *O. zibethicus* with 12 species of order Rodentia distributed in South Korea. The sequences accession number of the species used in phylogenetic analysis is shown in the figure.

The complete mitochondrial genome (GenBank Accession No. MT084805) was 16,350 base pairs in length and includes 13 protein-coding genes, 22 tRNA genes (ranging from 43 base pairs in tRNA-Pro to 75 base pairs in tRNA-Leu), two rRNA genes, and a control region. The order and pattern of gene arrangement of muskrat were the same with those of other species in order Rodentia. Its overall base contents are 33.9% for A, 23.8% for C, 12.5% for G and 29.8% for T, which means AT content bias (63.7%). All 13 PCGs initiated with ATN (ATA, ATC, ATG and ATT) codon, except for NADH dehydrogenase subunit I (ND1) started with GTG codon. Among 12 genes except for ND1, three PCGs (ND2, ND3 and ND5) start with ATT codon, and nine PCGs (ATP6, ATP8, COX1, COX2, COX3, CYTB, ND4, ND4L and ND6) start with ATG codon. Three PCGs (ND1, COX3, and ND4) terminate with T—, the incomplete stop codon. Ten PCGs (ND2, ND3, ND4L, ND5, ND6, COX1, COX2, CYTB, ATP6 and ATP8) terminate with TAA codon.

The phylogenetic tree using 13 species of order Rodentia is shown in Figure 1, and the muskrat *O. zibethicus* is closest to the *Myodes rutilus*. The mitogenome of *O. zibethicus* can provide useful information to understand the genetic evolution and the phylogenetic relationship of muskrat and relatives.

## Data Availability

The generated sequence data during this study is openly available in NCBI (National Center for Biotechnology Information) with GenBank Accession No. MT084805 (https://www.ncbi.nlm.nih.gov/nuccore/MT084805). Also, the data are available if required by the corresponding author (YK Kim).
